# Retroperitoneal Single-Port Robot-Assisted Nephroureterectomy with Bladder Cuff Excision: Initial Experience and Description of the Technique

**DOI:** 10.3390/jcm12186091

**Published:** 2023-09-21

**Authors:** Seokhwan Bang, Hyuk Jin Cho, U-Syn Ha, Ji Youl Lee, Sung-Hoo Hong

**Affiliations:** Department of Urology, Seoul St. Mary’s Hospital, College of Medicine, The Catholic University of Korea, Seoul 06591, Republic of Korea; drvion@gmail.com (S.B.); hyukjincho0403@gmail.com (H.J.C.); ushamd@catholic.ac.kr (U.-S.H.); uroljy@catholic.ac.kr (J.Y.L.)

**Keywords:** robotic surgery, single-port surgery, upper tract urothelial carcinoma, nephroureterectomy

## Abstract

Background: With the introduction of the single-port (SP) robot, surgery that was difficult to attempt is becoming possible. Nephroureterectomy (NUx) for upper tract urothelial carcinoma also seems to be able to attempt a retroperitoneal (RP) approach. Purpose: to investigate the feasibility of SP robotic RP NUx with bladder cuff excision. Design, setting, and participants: we sequentially analyzed 20 patients who underwent SP robot NUx from January 2021 to December 2022. Surgical procedure: all patients were diagnosed with upper tract urothelial carcinoma (UTUC) and were operated upon by a single expert using the da Vinci SP platform (Intuitive Surgical, Sunnyvale, CA, USA) with retroperitoneal approach. Results and limitations: A total of 20 patients underwent SP robotic NUx with bladder cuff excision. The mean age of patients was 69.45 ± 8.68 years, and the mean body mass index (BMI) was 25.37 ± 3.00 kg/m^2^. The mean tumor size was 2.42 ± 1.03 cm on a CT scan, with right-sided tumors in eight patients (40%) and left-sided tumors in 12 patients (60%). The median console time was 106 min and 40 s, and the expected blood loss was 122.50 ± 75.18 mL. Final pathology showed that all of the patients were diagnosed as having urothelial carcinoma; one patient was classified as Ta (5.00%), three patients were classified as T1 (15.00%), seven patients were classified as T2 (35.00%), eight patients were classified as T3 (40.00%), and one patient was classified as T4 (5.00%). None of these 20 patients showed any complications based on the Clavien–Dindo scale. Conclusions: SP robotic NUx using a retroperitoneal approach provides feasible perioperative and postoperative outcomes for UTUC.

## 1. Introduction

Urothelial carcinoma is the fourth most common cancer, and 5~10% of urothelial carcinomas are located in the upper tract [1,2]. The gold standard for the treatment of upper tract urothelial carcinoma is nephroureterectomy (NUx) with excision of the ipsilateral ureteral orifice and a bladder cuff [3,4].

The technique of laparoscopic NUx was first introduced in 1991 [5], and a clinical series was reported in 1993 [6]. Since then, the laparoscopic approach has become more common than the open approach, displaying no difference in oncological efficacy [7]. Afterwards, as surgical robots were developed, robot-assisted laparoscopic surgery gradually expanded and robots were also used in NUx [8].

**Figure 1:**
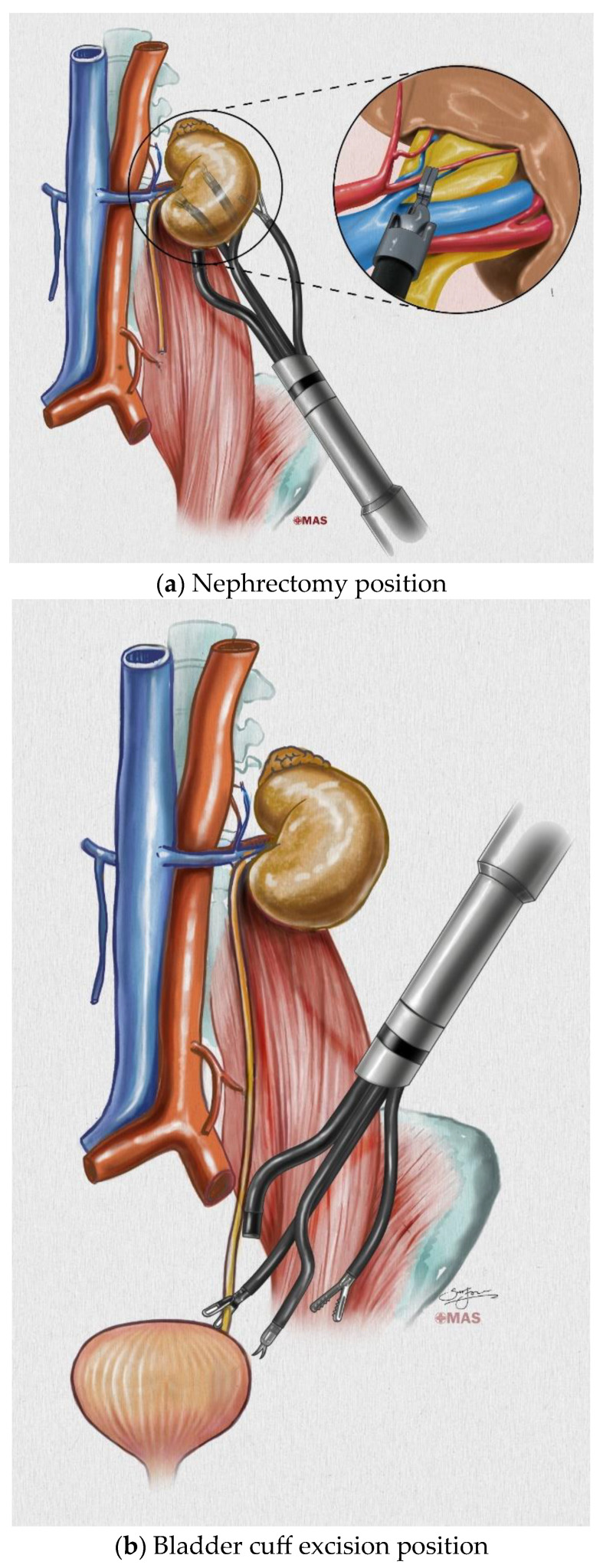
Schematics of retroperitoneal-approach nephroureterectomy with bladder cuff excision.

The kidney and ureter are organs located in the retroperitoneum; thus, the retroperitoneal (RP) approach has several advantages. The advantages include the following: the operation can be performed without touching the intestine, the tumor is limited to the retroperitoneum even if it is seeded, and it is loculated even if bleeding or urine leakage occurs. Therefore, in open NUx, the RP approach was considered the standard approach, but in the conventional robot, the port configuration required to access both the kidney and ureter was inappropriate and the distance between the ports was inadequate; hence, a transperitoneal (TP) approach was developed [9]. With the introduction of a single-port (SP) robot system, We recognized the possibility of the RP approach and attempted it (Figure 1).

Through this study, we attempted to prove the efficacy and safety of NUx performed via the RP approach using the SP platform.

## 2. Patients and Methods

### 2.1. Study Populations

We have been performing SP robot surgery since the SP robot was installed in the institution in September 2021, and SP NUx was performed with this technique from January 2022. After the beginning of January 2022, 20 patients were analyzed consecutively for one year. The operation was performed by a single expert, with experience of more than 1500 cases of robot surgery including 200 cases of SP robot surgery in an experienced center that performs over 100 MP robot-assisted RP partial nephrectomies annually. This study was reviewed by the IRB committee of Seoul St. Mary’s hospital (IRB No. KC22RISI0442).

### 2.2. Surgical Technique

**Figure 2:**
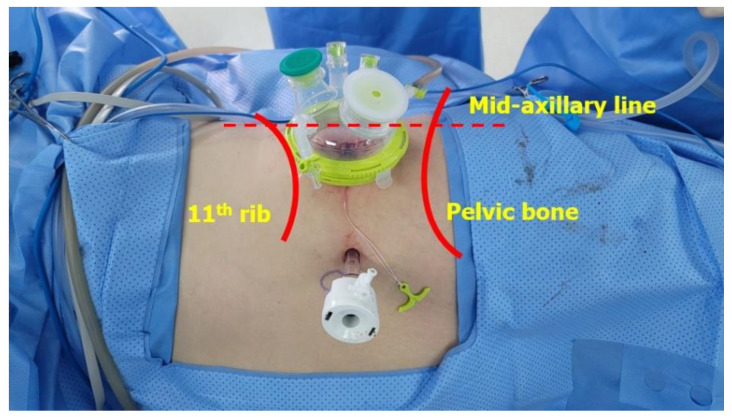
Docking port and trocar location of surgery.

**Figure 3:**
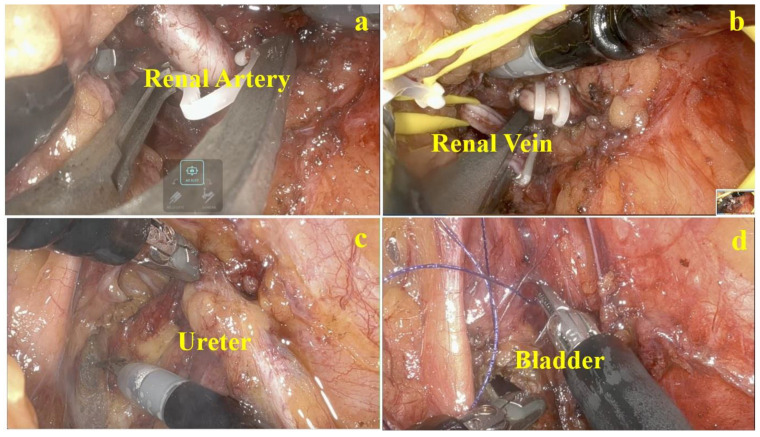
Surgery technique including (**a**) dividing of renal artery, (**b**) dividing of renal vein, (**c**) ureter tracking, and dissection (**d**) bladder cuff excision.

Under general anesthesia, the patient was placed in a lateral decubitus position and flexed to secure the flank space. The retroperitoneal space was secured using the well-established Gaur’s method [10]. A central point was marked in the space between the 11th rib and the iliac crest, and a 4 cm incision was made with the midclavicular line biased towards 1 cm anterior, considering bladder cuff excision. After dividing the muscle layer and confirming the retroperitoneal space, a space was created through ballooning twice towards the kidney and pelvis. We installed a 26 mm gel-port at this point. After inserting the docking port for the robot, a 12 mm assistant port was inserted, avoiding the peritoneum under the vision of robot camera (Figure 2). The Intuitive Da Vinci SP^®^ model was used for surgery. A pneumoretroperitoneum was created using a docking port with carbon dioxide. To perform radical nephrectomy first, the camera was positioned at 12 o’clock, monopolar scissors were positioned at 3 o’clock, Cadiere forceps were positioned at 9 o’clock and Maryland forceps were positioned at 6 o’clock in the case of a left-sided tumor. In the case of a right-sided tumor, the camera was positioned at 12 o’clock, monopolar scissors were positioned at 6 o’clock, Cadiere forceps were positioned at 3 o’clock and Maryland forceps were positioned at 9 o’clock. After removing the retroperitoneal fat as much as possible, Gerota’s fascia was opened, the kidney was lifted with Cadiere forceps, and the renal artery was approached. After dissection of the renal artery while checking the ureter path and the boundary of the aorta, the renal artery was divided using a Hem-o-lok^®^ and metal clip (Figure 3a). Then, the renal vein was dissected and divided in the same way (Figure 3b). After incision of Gerota’s fascia, the ureter was identified and clipped to prevent tumor seeding in case of a proximal ureter tumor or pelvic tumor.

The anterior portion of the kidney was isolated by carefully dissecting the gap between Gerota’s fascia and the peritoneum. Since it entered the posterior aspect of the kidney, it was sufficient to separate the retroperitoneal fat from the back. When the detachment was completed while saving the adrenal gland, the kidney was separated.

The robot was repositioned to enable bladder cuffing, but the position of the entire docking port was adjusted so that it could be directed towards the bladder beyond the psoas muscle without re-docking. When the direction was turned towards the patient’s pelvis, the camera position was still placed still at 12 o’clock; however, using the ‘Cobra mode’ (position the camera 30 degrees downward), the Cadiere forceps position was switched to 3 o’clock in the case of a left-sided tumor and 9 o’clock in the case of a right-sided tumor. The ureter was dissected up to the bladder while pulling with Cadiere forceps (Figure 3c). After confirming the detrusor muscle, monopolar scissors at the 3 o’clock position were converted to a needle driver, one stitch suture was performed using a barbed suture, and it was fixed with Cadiere forceps to prepare cuffing (Figure 3d).

Again, the needle driver was switched at 3 o’clock with scissors, an incision was made in the bladder to check the ureter orifice, and the cuff excision procedure was executed using Cadiere forceps to secure the sutures and prevent retroversion of the bladder. After that, the robot arm was switched back to a needle driver, and a continuous suture was performed with the previously placed needle to close the bladder. After suturing, 150 cc of saline was injected through a Foley catheter to check bladder closure. The specimen was extracted by extending the existing incision.

Patients were discharged on the 3rd or 4th day following surgery in accordance with the prescribed protocol. This procedure was implemented in alignment with the critical pathway and followed the same methodology as the conventional surgical approach. The Foley catheter was removed on the 10th day after surgery in an out-patient clinic.

### 2.3. Study Outcomes and Follow-Up

We reviewed all patients’ basic characteristics and perioperative outcomes. Patients’ postoperative complications were classified according to the Clavien–Dindo classification [11], and the length of hospital stay was calculated immediately after surgery. All patients received postoperative care following the same critical pathway. The Jackson-Pratt (JP) drain was removed, and the patient discharged on the 3rd or 4th day after surgery. The collected specimens were analyzed by the pathology department, and cell type and margin invasion were recorded separately.

Continuous variables were analyzed via the Mann–Whitney U test, and categorical variables were compared using a Chi-square test or Fisher’s exact test. All statistical analyses were conducted using SPSS (version 24, IBM, Chicago, IL, USA). A statistically significant value was defined as *p*-value < 0.05.

## 3. Results

### 3.1. Baseline Characteristics

The mean age of patients was 69.45 ± 8.68 years, and the mean BMI was 25.37 ± 3.00 kg/m^2^. The mean tumor size was 2.42 ± 1.03 cm on CT scan, with right-sided tumors in eight patients (40%) and left-sided tumors in 12 patients (60%). The tumor location was the renal pelvis in seven patients (35%), the proximal ureter in six patients (30%), the mid ureter in two patients (10%), and the distal ureter in three patients (15%). A preoperative CT scan of these 20 patients showed hydronephrosis in 11 patients and ureteritis in one patient. Upon preoperative cytology, urothelial carcinoma was suspected in five patients, atypical cells were found in eight patients, and normal findings were detected in seven patients (Table 1).

### 3.2. Surgical Safety and Perioperative Outcomes

The median operation time was 150.50 min, and the median console time was 106 min and 40 s. The expected blood loss was 122.50 ± 75.18 mL. No intraoperative complications were found during surgery. The median length of hospital stay was 4.5 days. Final pathology showed that all patients were diagnosed as having urothelial carcinoma; one patient was classified as Ta (5.00%), three patients were classified as T1 (15.00%), seven patients were classified as T2 (35.00%), eight patients were classified as T3 (40.00%), and one patient was classified as T4 (5.00%). Perirenal lymph node dissection was performed in 10 out of these 20 patients, and there were no positive nodes (Table 2).

### 3.3. Postoperative Outcomes

None of these 20 patients showed any complications based on the Clavien–Dindo scale. The average serum creatinine level in patients before surgery was 1.04 ± 0.331 mg/dL, the serum creatinine level immediately after surgery was 1.18 ± 0.261 mg/dL, and the serum creatinine level at the follow-up three months later was 1.19 ± 0.288 mg/dL. The results of conversion into estimated glomerular filtration rate (GFR) are summarized in Table 3. The average number of follow-up days for the 20 patients was 180.84 days, and only one patient had a tumor observed via cystoscopy, which was removed via a transurethral bladder tumor resection (TURBT) and diagnosed as a Ta low-grade urothelial carcinoma. Other than this, no recurrence was found on CT and cytology. Patients diagnosed as T3 following pathology after surgery were sent to the oncology department, and they were administered adjuvant chemotherapy.

## 4. Discussion

Through this study, we were able to conclude that NUx is feasible enough with the RP approach via the SP robot platform. Comparative studies are needed, but the results of surgery were satisfactory, and none of the patients had a positive surgical margin or postoperative complications.

From a technical point of view, when we make the incision line and insert a port, the mid-axillary line or a little more posterior line is more advantageous to access the kidney through the retroperitoneal approach; however, for distal ureter access, the anterior line is more advantageous than the mid-axillary line. Also, as described above, the part where the distal ureter is not visible due to the psoas muscle could be overcome by using the Cobra mode, which is a downward view of the camera.

Access to the retroperitoneum via an SP robot has is advantageous as no intraabdominal organs are encountered. In addition, in the case of men, there is the advantage that the vas does not need to be divided, and in the case of women, organs such as broad ligaments, ovaries, and the fallopian tube, can be preserved because of the approach being behind these structures. Another advantage is that no additional port insertion or re-docking is required for conversion from the kidney side to the pelvic side. The conversion is simple, and the change of direction alone is sufficient and can be completed within one minute. In the case of multiport robot surgery, the existing port site must be greatly extended or a new large incision must be made to remove the specimen, but in the case of SP surgery, only a small extension is required for the existing 4 cm incision. However, the surgical field of view is narrow; thus, a skilled operator is required.

To our knowledge, this is the first description of a retroperitoneal approach for nephroureterectomy using an SP robot. This introduction is meaningful as it secures a safer and minimally invasive technique by introducing a new surgical technique. Our surgical team has previously published a paper addressing the feasibility of SP robot-assisted RP partial nephrectomy [12], and this current study can be considered an extension of that research. We believe that the utilization of the SP robot can serve as a viable alternative to address the constraints posed by the confined surgical space in retroperitoneal surgery.

As the surgical procedure advances to the bladder cuff excision stage, and the robotic arm orients itself towards the patient’s leg, the assistant must execute actions in a mirrored configuration. While it does not constitute an exact mirror image, this setup introduces a misalignment between the motion direction and the visual perspective. To mitigate this issue, we adopted a positioning strategy where the assistant positions themselves with their back facing the patient. During the bladder cuff excision phase, the assistant adjusted their position by turning their back towards the camera, thereby aligning the camera axis more closely with the visual axis. However, it remains unequivocal that proficient surgical skills are imperative for effective surgical assistance.

We gathered a few tips from the 20 surgeries. One of them is the problem of a peritoneal opening that may occur during surgery. In the case of retroperitoneal access, when the peritoneum is opened, surgical gas leakage occurs and the surgical field of view is drastically reduced. If this happens, we repair the peritoneum using a Hem-o-lok^®^ clip and proceed with surgery. If peritoneal gas leakage persists after clipping, we can use 2 or 3 angio-needles to maintain the retroperitoneal space. In cases where repair is not possible because the open window is very wide, the peritoneum is completely opened and surgery can be performed, but this did not happen in our 20 cases.

Another advantage of retroperitoneal SP surgery is that it relates to fat removal. In the case of retroperitoneal surgery, the patient’s degree of obesity is one of the factors that has a great influence, and if the degree of obesity is high, the operation becomes difficult. In the case of multiport robotic surgery or laparoscopic surgery, even if the fat tissue is excised, it cannot be removed immediately; thus, it must be placed on one side of the narrow surgical space, which makes the surgery more difficult. However, in the case of SP surgery, the 40 mm docking port is opened for a while, and the cut fat can be removed immediately, which is very helpful for the operation.

As we performed surgery using this technique, we were able to recognize the limitations of this technique. This technique can easily dissect the periaortic or perirenal lymph node; however, there are limitations to pelvic lymph node dissection that should be considered in distal ureter cancer. Furthermore, when confronted with a sizable tumor located proximate to the ureterovesical junction, a broader excision may become imperative, potentially involving adhesions to adjacent organs, thereby rendering this surgical approach unsuitable for such cases. Therefore, it is recommended that the surgeon should keep this aspect in mind when considering the surgical approach or technique.

Of course, this study is a very early study of NUx using an SP robot, and there is a limitation as it was a retrospective study. The main aim of this manuscript is to introduce the surgical method and discuss its possibilities. In future research, more accurate analysis through prospective and randomized trials are needed. A comparative analysis with multiport robotic surgery or a transperitoneal approach is also warranted.

Also, since this study was conducted by a single operator, it was greatly affected by the operator’s individual ability. However, it is imperative that this aspect undergoes rigorous evaluation via a multi-center trial.

## 5. Conclusions

In this study, we demonstrate that RP SP robot-assisted NUx with bladder cuff excision is feasible. This approach holds particular promise for patients who have previously undergone intra-abdominal surgery, where access may be challenging.

## Figures and Tables

**Table 1:** Baseline characteristics.

All Patients				
*n*		20		
Mean age, years (range)		69.45	±	8.684
Gender (%)				
Male	9	45.00	%	
Female	11	55.00	%	
Mean BMI (kg/m^2^, range)		25.37	±	2.999
Tumor size on CT (cm)		2.42	±	1.030
Laterality				
Right	8	40.00	%	
Left	12	60.00	%	
Tumor location on CT				
Renal pelvis		7		35.00%
Proximal ureter		6		30.00%
Mid ureter		2		10.00%
Distal ureter		5		25.00%
Hydronephrosis on CT				
yes		11		55.00%
no		9		45.00%
Ureteritis on CT				
yes		1		5.00%
no		19		95.00%

BMI: Body mass index, CT: Computed tomography.

**Table 2:** Perioperative outcomes.

Operation Time (min, range)	150.5	±	29.074
Console time (min, range)	106.4	±	19.497
Estimated Blood Loss (mL)	122.50	±	75.18
T stage	Pathology		
T0			
Ta	1		(5.00%)
Tis			
T1	3		(15.00%)
T2	7		(35.00%)
T3	8		(40.00%)
T4	1		(5.00%)
N stage			
Nx	10		(50.00%)
N0	10		(50.00%)

**Table 3:** Postoperative outcomes.

pre op. creatinine	1.04	±	0.331
pre op. eGFR	68.10	±	19.849
post op. creatinine	1.18	±	0.261
post op. eGFR	55.80	±	9.956
3 month f/u creatinine	1.19	±	0.288
3 month f/u eGFR	55.60	±	10.753

Op.: operation, eGFR: estimated glomerular filtration rate.

## Data Availability

The data presented in this study are available on request from the corresponding author.
